# Effectiveness of a girls’ empowerment programme on early childbearing, marriage and school dropout among adolescent girls in rural Zambia: study protocol for a cluster randomized trial

**DOI:** 10.1186/s13063-016-1682-9

**Published:** 2016-12-09

**Authors:** Ingvild Fossgard Sandøy, Mweetwa Mudenda, Joseph Zulu, Ecloss Munsaka, Astrid Blystad, Mpundu C. Makasa, Ottar Mæstad, Bertil Tungodden, Choolwe Jacobs, Linda Kampata, Knut Fylkesnes, Joar Svanemyr, Karen Marie Moland, Richard Banda, Patrick Musonda

**Affiliations:** 1 Center for Intervention Science in Maternal and Child Health (CISMAC), Centre for International Health (CIH), University of Bergen, Bergen, Norway; 2 Centre for International Health, University of Bergen, Bergen, Norway; 3 Department of Global Public Health and Primary Care (IGS), University of Bergen, Bergen, Norway; 4 Department of Public Health, School of Medicine, University of Zambia, Lusaka, Zambia; 5 Department of Educational Psychology, School of Education, University of Zambia, Lusaka, Zambia; 6 Christian Michelsens Institute, Bergen, Norway; 7 Norwegian School of Economics, Bergen, Norway; 8 Central Statistical Office, Zambia, Lusaka, Zambia

**Keywords:** Adolescent pregnancy, Early marriage, School enrolment, Economic support, Community dialogue, Cash transfer, Sexual and reproductive health, School dropout, Poverty

## Abstract

**Background:**

Adolescent pregnancies pose a risk to the young mothers and their babies. In Zambia, 35% of young girls in rural areas have given birth by the age of 18 years. Pregnancy rates are particularly high among out-of-school girls. Poverty, low enrolment in secondary school, myths and community norms all contribute to early childbearing. This protocol describes a trial aiming to measure the effect on early childbearing rates in a rural Zambian context of (1) economic support to girls and their families, and (2) combining economic support with a community intervention to enhance knowledge about sexual and reproductive health and supportive community norms.

**Methods/design:**

This cluster randomized controlled trial (CRCT) will have three arms. The clusters are rural schools with surrounding communities. Approximately 4900 girls in grade 7 in 2016 will be recruited from 157 schools in 12 districts. In one intervention arm, participating girls and their guardians will be offered cash transfers and payment of school fees. In the second intervention arm, there will be both economic support and a community intervention. The interventions will be implemented for approximately 2 years. The final survey will be 4.5 years after recruitment. The primary outcomes will be “incidence of births within 8 months of the end of the intervention period”, “incidence of births before girls’ 18th birthday” and “proportion of girls who sit for the grade 9 exam”. Final survey interviewers will be unaware of the intervention status of respondents. Analysis will be by intention-to-treat and adjusted for cluster design and confounders. Qualitative process evaluation will be conducted.

**Discussion:**

This is the first CRCT to measure the effect of combining economic support with a community intervention to prevent adolescent childbearing in a low- or middle-income country. We have designed a programme that will be sustainable and feasible to scale up. The findings will be relevant for programmes for adolescent reproductive health in Zambia and similar contexts.

**Trial registration:**

ISRCTN registry: ISRCTN12727868, (4 March 2016).

**Electronic supplementary material:**

The online version of this article (doi:10.1186/s13063-016-1682-9) contains supplementary material, which is available to authorized users.

## Background

Approximately 7.3 million girls below age 18 give birth in low- and middle-income countries (LMICs) every year [1]. Maternal complications are estimated to be the fourth most common cause of death in girls aged 15–19 in LMICs [2], and the risks of prematurity and low birth weight are high in adolescent pregnancies [3–5], with consequent higher morbidity and mortality [4, 6–10]. The younger the mother is, the higher is the risk of complications both for her and the child [3], and childbearing before age 16 is of particular concern [4, 6–8].

Early pregnancy is often associated with early marriage and school dropout, and poverty contributes to all three. Observational studies from low-income countries indicate that young women who quit school early are more likely to marry and become pregnant earlier than those who stay in school [11–15]. Increased schooling has also been associated with better health of women and their children [16, 17]. In the last decades primary school enrolment has increased significantly in many poor countries. However, enrolment at secondary level is much lower than at primary level in most LMICs, particularly for girls [18]. This may be due to limited availability of and longer distances to school, higher fees, or to early marriage or pregnancy. Moreover, there may be a preference to support boys’ education rather than girls’ [19]. In many societies marrying off a girl may be regarded as better to secure her future than schooling, and the bride-price paid to the girl’s family may be an important source of income. Once a girl is married, she is expected to start childbearing. Moreover, where access to cash is severely limited, many unmarried adolescent girls engage in sexual relationships, even if not socially acceptable, to receive gifts and cash [20–22].

We did a systematic search^1^ in 16 databases^2^ for randomized controlled trials (RCTs) in LMICs that assessed interventions targeting adolescent childbearing and marriage. Recent systematic reviews on adolescent pregnancy [23, 24] and childbearing [25] and early marriage [26, 27] were also examined for relevant original articles. We focused on trials that included girls younger than 18. Three trials examined the effects of support to girls to reduce the cost of schooling. In Kenya, girls provided with free school uniforms were less likely to drop out before completing primary school, and proxy reports by classmates indicated reduced risks of early marriage and childbearing [28]. A trial in Zimbabwe found that a programme targeting orphan girls, offering payment of school fees and free uniform, led to an 80% reduction in school dropout, and 60% reduction in marriage rates in the next 2 years [29]. A trial in Malawi found that payment of school fees combined with small cash transfers to adolescent girls and their families resulted in lower prevalence of human immunodeficiency virus (HIV) and Herpes simplex virus type 2 (HSV-2) in the next 18 months among girls enrolled in school at baseline [30]. The trial also found that unconditional cash transfers led to reduced marriage and pregnancy rates whereas no significant change was seen in these outcomes in the group provided with cash transfers conditional on school attendance [31]. Quasi-experimental evaluations of unconditional and conditional cash transfer programmes in Kenya [32], Pakistan, [33] and Mexico [34] have also found effects on adolescent childbearing and/or marriage. More recently, two RCTs on the effects of conditional cash transfer programmes on HIV have been conducted in South Africa [35]. They found no effect on HIV incidence [22]. Thus effects of cash transfers appear to be context dependent [24], and there is a need for further evaluations of such programmes in new settings, alone and in combination with other interventions [22].

Comprehensive sexual and reproductive health education (CSRHE) programmes can equip adolescents with knowledge and skills to prevent unwanted pregnancy even if they are sexually active. Reviews of evaluations of sexual and reproductive health education programmes worldwide indicate that effective curricula are intensive [36], include student active teaching [37], and incorporate discussions around gender and power dynamics [38]. Our systematic literature review of RCTs identified five cluster randomized controlled trials (CRCTs) from LMICs that studied the impact of sexual and reproductive health (SRH) education programmes on early childbearing and marriage. The findings were mixed: a CRCT in Kenya found that pupils exposed to a curriculum informing them of the higher risk of HIV infection associated with having older sexual partners, had 28% lower pregnancy rates 12 months later [39], whereas training of teachers in the national HIV/AIDS curriculum promoting sexual abstinence until marriage, indicated no effect on adolescent childbearing after 2, 3, 5 and 7 years [28, 39, 40]. This is in line with a systematic review of randomized studies assessing abstinence-only programmes in the US, finding no protective effects on self-reported sexual behaviour, sexually transmitted infection (STI) diagnosis, or pregnancies [41]. In Uganda, a CRCT assessing a combination of a life skills programme (which included comprehensive sexual and reproductive health education) and vocational skills training, found lower probability of adolescent girls in the intervention arm having children after 2 years compared to the control [42]. In South Africa, a CRCT evaluated the impact of a 50-hour interactive HIV prevention programme focusing on SRH and gender relations was evaluated. The intervention was delivered over 8 weeks to girls and boys (in- and out-of-school) aged 16–19. After 2 years the proportion of girls who were pregnant tended to be higher in the intervention arm (OR 1.45; 0.92–2.28), there was no difference in HIV incidence between the two arms, whereas the HSV-2 incidence was 33% lower in the intervention than control group [43]. Another CRCT in South Africa studied a comprehensive teenage pregnancy prevention programme with 12 weekly sessions among pupils in grade 8 and found no impact on pregnancy rates after 8 months [44]. In Tanzania, a CRCT examined the effects of an intervention with the following four components: (1) a teacher-led, SRH school programme in grades 5–7; (2) training and supervision of health workers in youth-friendly health services; (3) community-based condom promotion and distribution by adolescents; and (4) 1-week mobilization in each community and annual youth health weeks. An evaluation 3 years after the start of the intervention found effects on SRH knowledge and self-reported condom use among the youths, but no impact on the incidence of HIV, HSV-2 or pregnancy despite high coverage and high quality of the implementation [45]. Qualitative evaluation during the first years of the intervention indicated that adolescents found it difficult to use the new knowledge and skills because of attitudes and practices in the community. The authors suggest this may imply that SRH education may not be effective on its own, but needs to be combined with efforts to “address broader sexual norms” and future aspirations [46–48].

The need to find ways to prevent early marriage and pregnancy is high on the political agenda in Zambia. As many as 35% of young rural 18-year-old girls have given birth, and the median age at marriage was 18 years in 2013/2014 [49]. According to the 2010 Zambia Census of Population and Housing (ZCPH), the pregnancy-related mortality ratio among girls aged 15–19 years is 80% higher than among those aged 20–24 [50]. Adolescent childbearing rates are much higher in rural than urban areas, and higher among girls who are out-of-school compared to those still attending (36% vs 5% at age 17 in rural areas) (unpublished data from 2010 ZCPH).

In preparation for this trial, we conducted formative research to explore contributors to early childbearing, marriage and school dropout. In-depth interviews (IDIs) and focus group discussions (FGDs) were conducted with girls, parents, teachers, health workers and community leaders in December 2014 and January 2015. Poverty was raised as a primary reason for early childbearing, early marriage and school dropout; girls mentioned a desire for gifts/money as an important reason for engaging in sexual relations; and parents put pressure on their daughters to get married to secure their daughters economically and to obtain the bride price. Moreover, many families could not afford uniforms or school fees at secondary school level. Other factors leading to early pregnancy were misconceptions linked to contraception, e.g. that girls could end up as infertile if they use hormonal contraception before their first pregnancy, social barriers to seeking contraception, and school dropout. Girls who have dropped out of school were regarded as ready for marriage after menarche. The SRH curriculum in Zambian schools is comprehensive on paper [51], but the interviews with teachers indicated that in practice a high proportion of them tend to focus on sexual abstinence as the only way to avoid pregnancy or sexually transmitted infections (STIs), including HIV. Hence misconceptions about modern contraceptives can prevail. Pregnancy was also described as leading to school dropout and marriage, although some girls reenter school after having given birth.

Since early pregnancy and marriage in Zambia have multiple causes, our contextual assessment implies that it is likely that a programme that targets several contributing factors will have a greater effect than a single focus intervention. This is in line with the existing literature, which has increasingly recognized that multicomponent interventions are needed to achieve a substantial impact on complex issues such as adolescent childbearing, early marriage and school dropout [52–55]. Further, as long as a high proportion of girls never enrol in secondary school, preventing early marriage and childbearing also needs to reach out-of-school girls. Informed by our literature review, the formative research, discussions with key stakeholders such as the Ministry of General Education (MoGE), the Ministry of Community Development and Mother and Child Health (MCDMCH) and local stakeholders, we developed an intervention package that targets what we identified as the main causes of early pregnancy: (1) an economic component targeting poverty and school dropout, with the aim to increase school attendance and secondary school enrolment, and to reduce parental pressure for early marriage and girls’ dependence on having a boyfriend to receive basic goods; and (2) a community component, including a youth club, that aims to enhance SRH knowledge and skills, and perceived supportive community norms regarding pursuit of educ﻿atio﻿n and﻿ postponement of pregnancy and marriage. Our hypothesis is that the timing of support is important, and payment of school fees, which reduces the cost of schooling, may be decisive at stages when children approach transition points in the education system, e.g. between primary and secondary school, when costs would otherwise increase.

### Objectives

Primary objectives:

1. To measure the effectiveness of a combined economic and community intervention on childbearing within 8 months of the end of the intervention period.
2. To measure the effectiveness of economic support alone and of a combined economic and community intervention on childbearing before the 18th birthday among girls.
3. To measure the effectiveness of economic support alone and of a combined economic and community intervention on the proportion of girls who sit for the grade 9 exam.

Secondary objectives:

To measure the effectiveness among girls of economic support alone and of a combined economic and community intervention on:

1. Pregnancy and childbearing within 2 years of the end of the intervention period
2. Pregnancy and childbearing before the 16th birthday
3. Pregnancy before the 18th birthday
4. Marriage before the 16th and 18th birthday
5. Socioeconomic inequality in childbearing and marriage before the 18th birthday
6. Enrolment in grade 8 and 10, school attendance, and grade 9 exam scores
7. Socioeconomic inequality in participation in the grade 9 exam
8. Knowledge and experiences related to sexual and reproductive health, including modern contraceptives
9. Attitudes and perceived community norms related to: the value of educating girls, early marriage, modern contraceptive use, and adolescent pregnancy
10. Employment status

## Methods

### Study design

**Fig. 1:**
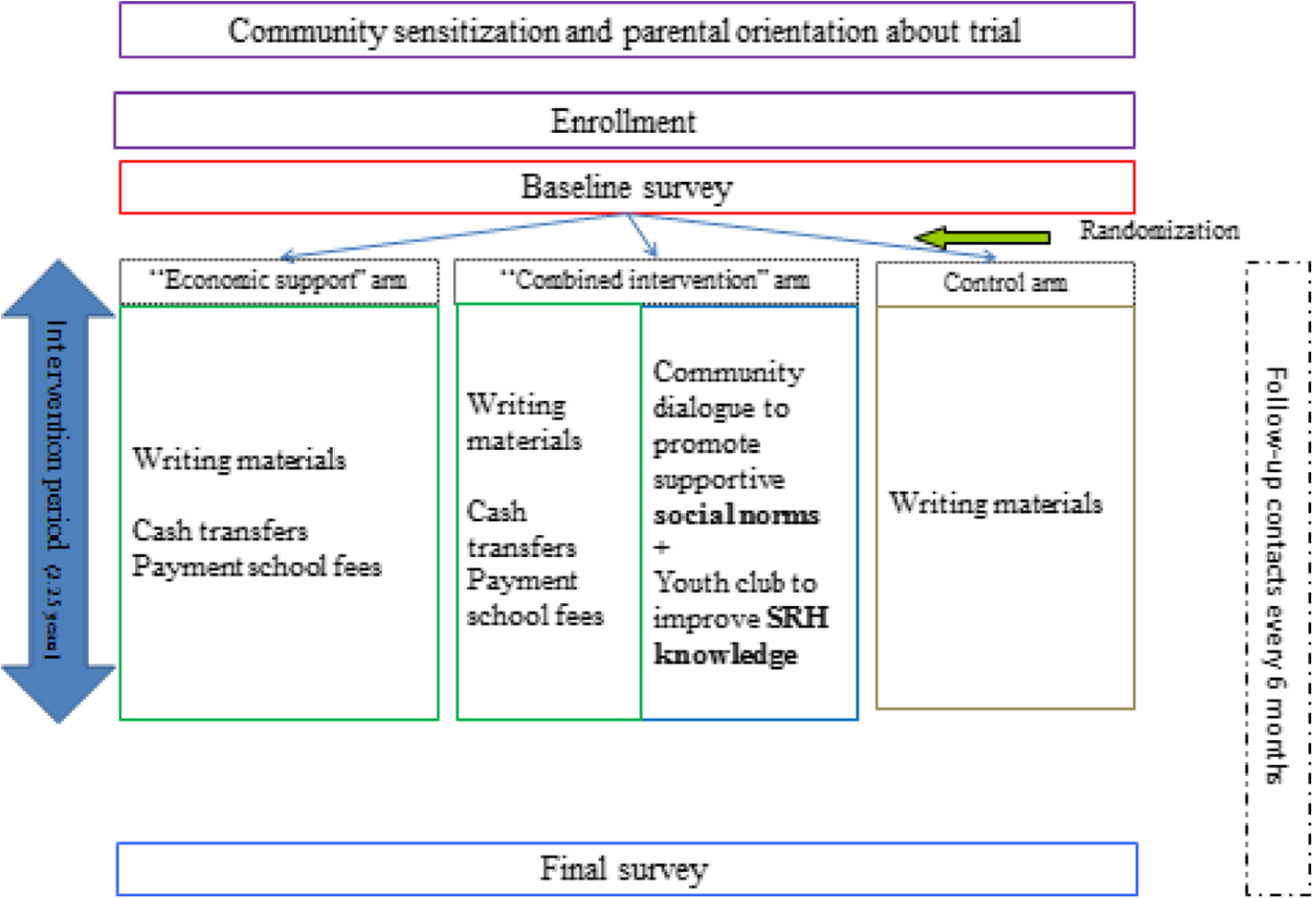
Flow chart of trial

The intervention packages will be examined in a cluster randomized controlled trial with two intervention arms and one control arm (Fig. 1). The randomization units will be basic schools and their surrounding communities. (Basic schools offer grades 1–9. Primary school in Zambia comprises grade 1–7 and grades 8 and 9 are referred to as junior secondary school.) The selected schools are at least 8 km apart from each other.

### Study setting and participant population

The participants will be girls enrolled in grade 7 in 2016 in rural basic schools in 12 districts in Zambia: Kalomo, Choma, Pemba, Monze, Mazabuka, Chikankata, Chisamba, Chibombo, Kabwe, Kapiri Mposhi, Mkushi, and Luano. These districts were selected as they have medium school dropout rates, and adolescent marriage and childbearing are common. All girls enrolled in grade 7, including anyone who is already married or has children, will be eligible to participate. Girls who drop out of school after they have been recruited will still be followed up and can continue to receive the interventions.

### Community sensitization and acceptance

To achieve community acceptance, chiefs, headmen, religious - and informal - leaders, headmasters and Parent Teacher Association (PTA) committee members were oriented and asked to support the trial activities before the recruitment was initiated. Local radio has also been used for community sensitization. Communities and schools were informed t﻿hat any individual school in which > 15% of the girls did not assent would not be included in the trial.

### Interventions

The interventions will be launched in September 2016 and will last for 27 months, until November 2018 (the end of the academic year when the girls who attend school are expected to complete grade 9). The intervention may be extended one more year, given supplementary funding. In all the study arms, girls will be offered writing materials (exercise books, pencils and pens) as an incentive to participate. Apart from this, only standard school and health services will be offered in the control arm.

#### Economic support

In the intervention arms, girls and their parents/guardians will be offered economic support, consisting of a monthly cash transfer for the girl (ZMW 30), an annual cash grant to her parents/guardians (ZMW 350/year) and payment of school fees for girls who enrol in grade 8 and 9 (up to ZMW 500 per term). The support package targets the key actors in the decisions leading to early pregnancy and marriage. Cash transfers target the poverty dimension, by making it somewhat less urgent for the guardians that the girl gets married and for the girl to receive gifts from a boyfriend.

The payment of school fees will be made directly to the school bank account for girls who get a place in grade 8 and 9. The money for the girls and the guardians will be disbursed by a cash transfer committee consisting of a teacher and two parents from the PTA committee. At least two of the cash transfer committee members will be present during disbursement to witness that the right persons receive the cash. Girls will also be asked in every follow-up contact how much money they have received, and all participants will be encouraged to contact the study team if they do not receive the right amount.

There will be no age limit for the economic support for girls who are in school, but for girls who drop out of school, it will stop after the 18th birthday. The economic support will also be discontinued for girls who do not participate in the follow-up contacts.

#### Community dialogue

The second intervention arm will combine the economic support with a community-oriented intervention consisting of (1) community and parent meetings employing a community dialogue approach in promoting supportive community norms around education for girls and postponement of early marriage and early childbearing; and (2) establishment of youth clubs in order to provide comprehensive sexual and reproductive health education among in- and out-of-school adolescent girls and boys.

Girls participating in the trial and boys who attend grade 7 in 2016 in the randomly selected schools will be invited to participate in a youth club every fortnight during the school terms, and they will all be welcome to continue in the youth club even if they quit school. The meetings will include interactive discussions on education, early marriage, the risks of early pregnancy, gender roles, and sexual and reproductive health, including myths around modern contraceptives. We will test a model where a teacher is linked with a community health assistant (CHAs) or a community health worker (CHW) to run the youth club together. Meetings will be held to inform parents about the content of the youth club sessions. Before the intervention is launched, the selected teachers and CHAs/CHWs will be given a 5-day training which will focus on the SRH curriculum, facilitation techniques and approaches to community mobilization. Refresher training will be held halfway during the intervention period. In addition, orientation meetings will be held to inform other healthcare workers in the catchment area of the schools about the project and the importance of providing youth-friendly health services.

For each combined intervention cluster, two young (<20 years) unmarried women from the local community will be selected as youth peer educators. The role of the peer educators will be to mobilize girls and boys to come to youth club meetings.

Meetings with the community at large will be organized bimonthly. These meetings will be conducted using a dialogue approach [56] and will discuss topics such as the value of education, and the risks and benefits of early childbearing. Films or role plays will be used to start discussions.

### Outcomes

The outcomes, measurement tools, measurement times and validation tools are listed in Table 1.

**Table 1:** Outcomes, measurement tools, measurement times and validation tools

		*Measurement tool*	*Months from recruitment to measurement*	*Validation*
*Primary outcomes measures*	Incidence of births within 8 months of the end of the intervention period	Follow-up contact questionnaire (FupQ)	42–44	FinQ 54–56 months after recruitment
	Incidence of births before girls’ 18th birthday	Final questionnaire (FinQ)	54–56	
	Proportion of girls who sit for the grade 9 exam	FinQ	54–56	Exam registers from District Education Board Secretary (DEBS) 54–56 months after recruitment
*Secondary outcome measures*	Pregnancy and childbearing			
	Incidence of pregnancies among girls within 2 years of the end of the intervention period	FinQ	54–56	
	Incidence of births among girls within 2 years of the end of the intervention period	FinQ	54–56	
	Incidence of pregnancies before girls’ 16th birthday	FinQ	54–56	FupQ 24–48 months after recruitment
	Incidence of births before girls’ 16th birthday	FinQ	54–56	FupQ 24–48 months after recruitment
	Incidence of pregnancies before girls’ 18th birthday	FinQ	54–56	
	Socioeconomic inequality in proportion of girls who have given birth before their 18th birthday	FinQ	54–56	
	Marriage			
	Proportion of girls that are married and/or cohabiting before their 16th birthday	FinQ	54–56	FupQ 6-48 months after recruitment
	Proportion of girls that are married and/or cohabiting before their 18th birthday	FinQ	54–56	
	Socioeconomic inequality in proportion of girls that are married/cohabiting before their 18th birthday	FinQ	54–56	
	School-related			
	Proportion of girls who enrol in grade 8	FupQ	12–14	School registers
	Socioeconomic inequality in participation in the grade 9 exam among girls	FinQ	54–56	Exam registers December 2018 and 2020
	Proportion of girls who enrol in grade 10	FupQ	36–38	School registers
	Average examination scores of girls from grade 9 in English, mathematics and science	Exam results from District Education Board Secretary (DEBS)	Dec 2018	
	School attendance of girls in grade 8	School registers	Dec 2018	FupQ 12–14, and 18–20 months after recruitment
	School attendance of girls in grade 9	School registers	Dec 2018	FupQ 24–26 and 30–32 months after recruitment
	Other reproductive health outcomes			
	Proportion of adolescent girls who have been sexually active in last 4 weeks	FupQ	30-32	
	Proportion of adolescent girls currently using modern contraceptives	FupQ	30-32	
	Knowledge of modern contraceptives among adolescent girls	FupQ	30-32	
	Perceived community norms			
	Perceived community norms regarding modern contraceptive use among unmarried adolescent girls	FupQ	30-32	
	Perceived community norms regarding early marriage among girls	FupQ	30-32	
	Perceived community norms regarding adolescent pregnancy among girls	FupQ	30-32	
	Perceived community norms regarding education among girls	FupQ	30-32	
	Other			
	Proportion of girls currently employed or self-employed	FinQ	54–56

**Table 2:** Assumptions for sample size required to measure the primary outcome “incidence of births before girls’ 18th birthday”

Parameter	Assumed level	Comment
Incidence of births before girls’ 18th birthday	0.08	We assume that 27% of girls in the control arm will have given birth before their 18th birthday. This corresponds to an average incidence rate of (27%-3%) = 8% per year over the average 3-year period (from the time the average age is 15).
Effectiveness of combined intervention vs control	−40%	i.e. the incidence in combined intervention arm assumed to be 0.048
Effectiveness (i.e. (1-RR) × 100 of economic intervention vs control	−25%	i.e. the incidence in economic intervention arm assumed to be 0.06
Effectiveness of combined intervention vs economic intervention	−20%	The combined intervention will offer [1-(0.0.048/0.06)], i.e. 20% more relative protection than the economic intervention alone.
Cluster size	28	Average number of girls in grade 7 in the selected schools is 31. If we assume that up to 10% may be lost to follow-up by the time of measuring the outcome, the average cluster size will be 28
Person years per cluster	84	If 28 participants are followed up for 3 years on average, person years per cluster are 84.
K	0.15	The ICC was 0.00737 for “ever pregnant” after the intervention period in the cash transfer trial in Malawi (estimate obtained from Sarah Baird). This corresponds to k = 0.15 when the total proportion who have given birth by this time is 0.27.
Z_1_	1.96	
Power for comparison of economic intervention vs combined intervention	70%	We need 63 clusters in each of the intervention arms to have 70% power to detect the assumed difference
Power for comparison of economic intervention vs control	80%	We need 39 clusters in each arm to have 80% power to detect the assumed difference. The PASS power calculator for incidence rates does not allow for unequal trial arms, but the PASS function for proportions indicates that 63 economic and 31 control will give slightly higher power
Power for comparison of combined intervention vs control	>95%	We need 23 clusters in each arm to have 95% power to detect the assumed difference.

### Sample size

We used PASS 14 (NCSS Statistical Software, Kaysville, UT, USA) to calculate sample size required for a cluster randomized trial with the primary outcomes “incidence of births within 8 months of the end of the intervention period”, “incidence of births before girls’ 18th birthday” and “proportion of girls who sit for the grade 9 exam”.

We used the 2010 census estimates of the percentages reporting ever giving birth in the study districts to estimate the incidence of childbearing in the control arm: 2% at the average age of 14.5 years, 4% at 15.5 years, 9.5% at 16.5 years, 22% at 17.5 years, and 35% at 18.5 years. The interventions will not affect childbearing until approximately 9 months after the start of the intervention, when the average age will be 15. We assume that 3% of the girls will have given birth before any effects of the interventions can be seen. The other assumptions for the sample sizes required to detect differences for the primary outcomes “incidence of births before girls’ 18th birthday” and “incidence of births within 8 months of the end of the intervention period” are presented in Tables 2 and 3. We find (using two-sided tests) that with 63 clusters in each of the intervention arms and 31 in the control arm we will have 70% power to detect the assumed difference between the economic and the combined interventions, 80% power for the comparison of the economic intervention versus the control arm, and > 95% power to detect the assumed difference between the combined intervention versus the control arm for the outcome “incidence of births before girls’ 18th birthday”. For the outcome “incidence of births within 8 months of the end of the intervention period” we will have > 90% power for the comparison of the combined intervention versus the control group.

The percentage of girls enrolled in grade 7 who sat for the grade 9 exam was approximately 50% in 2012. In 2015 the Ministry of General Education (MoGE) removed the previous entry requirements for grade 8 in Central province and all pupils who sit for the grade 7 exam in Chibombo, Chisamba, Kapiri Mposhi, Kabwe, Mkushi and Luano will be admitted to grade 8. To be enrolled they will have to pay school fees. The percentage who enrols in grade 8 is very likely to increase due to this policy change. This policy has not been introduced in Southern province (where the districts Kalomo, Choma, Pemba, Monze, Mazabuka and Chikankata are located). We have assumed that the percentage will be 70% overall in the control arm. See Table 4 for the other assumptions. With 30 clusters in each arm, we will have 95% power or more for each of the three comparisons.

**Table 3:** Assumptions for sample size required to measure the primary outcome “incidence of births within 8 months of the end of the intervention period”

Parameter	Assumed level	Comment
Incidence of births in control group	0.06	Eight months after the end of the intervention period the girls will be on average 17.1 years and we assume that 15% of them will have given birth. This corresponds to an average incidence rate of (15%-3%)/2 = 6% over this 2-year period.
Effectiveness of combined intervention vs control	−40%	i.e. incidence in combined intervention arm assumed to be 0.036
Cluster size	28	See assumptions for the outcome “incidence of births before girls’ 18th birthday”
Person years per cluster	56	If 28 participants are followed up for 2 years, there will be 56 person years
k	0.20	The ICC was 0.00737 for “ever pregnant” after the intervention period in the cash transfer trial in Malawi. This corresponds to k = 0.20 when the total proportion who have given birth by this time is 0.15
Z_1_ (acceptable alpha error level)	1.96	
Power for comparison of combined intervention vs control	90%	We need 36 combined clusters vs 36 control clusters to have 90% power detect the assumed difference. The PASS function for proportions indicates that with 63 combined clusters, we will have > 90% power with 30 control clusters.

**Table 4:** Assumptions for sample size required to measure the primary outcome “proportion of girls who sit for the grade 9 exam”

Parameter	Assumed level	Comment
Proportion of girls who sit for the grade 9 exam in control arm	0.70	
Effectiveness of combined intervention vs control	+26.5%	i.e. proportion completing in combined intervention arm assumed to be 0.886
Effectiveness (i.e. (1-RR) × 100 of economic intervention vs control	+15%	i.e. proportion completing in economic intervention arm assumed to be 0.805
Effectiveness of combined intervention vs economic intervention	+10%	The combined intervention will offer a [1 + (0.886/0.805)], i.e. 10% relative increase compared to the economic intervention alone.
Cluster size	28	See assumptions for the outcome “incidence of births before girls’ 18th birthday”
ICC	0.02	We have no information on the ICC for this outcome but have assumed it to be higher than for pregnancy
Z_1_	1.96	
Power for comparison of economic intervention vs combined intervention	95%	We need 29 clusters in each of the intervention arms to have 95% power to detect the assumed difference
Power for comparison of economic intervention vs control	95%	We need 24 clusters in each of the arms to have 95% power to detect the assumed difference
Power for comparison of combined intervention vs control	>95%	We need 7 clusters in each arm to have 95% power to detect the assumed difference

**Table 5:** Data collection elements

Activity	Description
Baseline survey	Trained research assistants conduct baseline face-to-face interviews with girls at school immediately after recruitment. The structured questionnaires cover questions on date of birth (Additional file 4), marital status, previous childbearing, household assets, perceived community norms related to education, early marriage and childbearing, and knowledge about SRH and contraceptives (Additional file 5). Mobile phone numbers of participating girls (if any), parents/guardians and close relatives or neighbours are recorded for use in the follow-up interviews (Additional files 4 and 6). Brief interviews are also done with the girls’ guardians about the educational attainment of the girls’ parents and head of household (Additional file 6).
Follow-up contacts	All the girls in the trial will be contacted every 6 months via phone to update contact information and ask questions about school attendance, employment, marital status and childbearing. Girls in the intervention arms will be asked whether they have received the cash transfers and/or participated in the youth club meetings. To monitor adverse events, girls will also be asked about hospitalizations and whether they have experienced problems due to their participation in the trial. If the girl cannot be reached via phone, field-based research assistants will attempt to meet and interview her (Additional files 7, 8, and 9).
Qualitative process evaluation	IDIs and FGDs will be conducted to explore experiences with the various intervention components of purposively selected girls, boys, parents/guardians, and other community members.
Quantitative process evaluation	We will collect attendance lists from each youth club meeting and reports with counts of how many persons show up at all parent – and community– meetings. Information on who has received cash transfers and payment of school fees will be recorded. We will collect data on school performance, school attendance and exam results of all study participants from school registers.
Final follow-up survey	A phone-based final survey will be conducted at the end of 2020 when the majority of the girls will be above 18 years. The questionnaire will include measurements of the primary and secondary outcomes. If the girl cannot be reached via phone, field-based research assistants will attempt to meet her face-to-face. If additional funding is secured we will consider doing these interviews face-to-face instead.

*FGD* focus group discussion, *IDI* in-depth interview, *SRH* sexual and reproductive health

Taking the largest of these sample sizes, we need 63 clusters in each of the intervention arms. Since we expect larger differences between each of the intervention arms and the control arm than between the two intervention arms themselves we can reduce the total number of clusters by allowing for a lower sample size in the control arm. Thus we will include at least 63 economic intervention clusters, 63 combined intervention clusters and 31 control clusters, i.e. 157 clusters with a total of 31 × 157 = 4867 or approximately 4900 girls.

### Recruitment

**Fig. 2:**
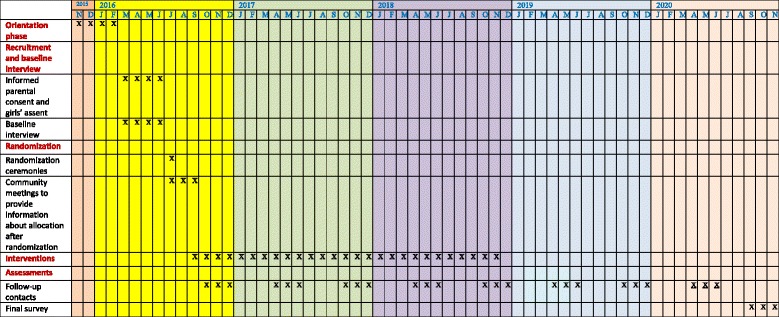
SPIRIT figure. Schedule of enrolment, interventions, and assessments

The recruitment started in March and will be completed in July 2016 (Fig. 2: SPIRIT figure). The parents/guardians of girls in grade 7 are invited to an information meeting. If their daughter is < 18 years, they are asked to give consent to her participation in the trial (Additional file 1). After the consent is obtained, girls are informed and asked to assent by a research assistant (Additional file 2). Any girl aged ≥ 18 years is informed directly and asked to consent (Additional file 3). Those who consent/assent and participate in the baseline survey interview are enrolled.

### Randomization and masking

Randomization will take place in July 2016, after recruitment is completed. We will organize six randomization ceremonies, each for two districts, where the schools will be stratified by district and randomly allocated to the three arms. Before each ceremony, 1000 allocations will be computer-generated by an independent scientist from the Centre for Interventions Science in Maternal and Child health (CISMAC), and each allocation will be numbered. Officials from the study districts, chiefs, head teachers and PTA members of the trial schools will be invited to be present. Community members will be welcome too. Tickets with numbers corresponding to a specific allocation will be drawn from a box.

There will be no blinding of participants, but the team doing the final survey will be independent from the intervention delivery and they will be unaware of the intervention status of respondents. Biannual follow-up contacts with the participants and the final surveys will be conducted by research assistants who are independent from the intervention implementation team.

### Data collection

All the tools have been translated to Tonga, Nyanja, Bemba and Lenje, the major local languages in the study districts, and then back-translated to ensure that the content is maintained. Interviews are conducted in English or one of these four local languages, depending on the preference of the participant. The main data collection tools are described in Table 5.

We will collect information about the location and size of schools that are excluded due to suboptimal participation.

### Data management and quality assurance

All the quantitative tools have been piloted to ensure that the questions and the translations are relevant and comprehensible. Data from interviews will be captured electronically using tablets and CommCare (https://www.commcarehq.org/home/) as our data management software. The forms have inbuilt check-and-skip rules to minimize data entry errors.

Names and telephone numbers and addresses will not be recorded in the same forms as sensitive data. Each participant will be given a unique identifier, and this number will be used when storing forms. Only the Data Manager, the Principal Investigator and the Co-Principal Investigator will have access to the personal identifiers. All data will be saved on password-protected computers and tablets. The database is hosted by Dimagi (http://www.dimagi.com/), and weekly downloads of data are done to a secure server owned by the University of Bergen. When the trial is completed, all personal identifiers will be deleted.

In the qualitative process evaluation, we will seek permission to audio record the individual interviews and focus group discussions using digital recorders. The recordings will be transcribed and translated verbatim. A senior researcher will review the transcripts to ensure that they contain word by word transcriptions and translations that retain original meanings, with a particular caution to retain culturally embedded content.

### Statistical methods

The data will be analysed with Stata 14 (StataCorp, College Station, TX, USA) software. The outcomes will be captured in time-to-event, binary and continuous variables. Descriptive statistics will be used to describe continuous and categorical variables. We will compare outcomes between the three arms: the economic arm versus the control arm, the combined arm versus the control arm, and the economic arm versus the combined intervention arm. Analyses will be by intention-to-treat (ITT).

The childbearing outcomes will be measured based on the participants’ responses to the questions “Have you ever given birth to a baby who was born alive?”, “Have you ever given birth to a baby who was born dead?”, “If yes, on which date and which year did you give birth?” and “How many months pregnant were you when you gave birth?”. We will count stillbirths after 6 months or 28 weeks pregnancy as births. Pregnancies conceived before the intervention starts will be excluded from the analysis of effects on childbearing and pregnancy.

For the incidence measures of childbearing, survival analysis using Cox regression will be employed. Time-on-study will be used as the timescale when measuring outcomes occurring within 8 months or 2 years of the end of the intervention period, and age will be the timescale when measuring outcomes before the 16th and 18th birthdays. Proportions will be compared using generalized estimating equations (GEE) with a log link. The ICC will be reported for all primary and secondary outcomes. Any remaining imbalances of predictors of the outcomes after randomization will be adjusted for in the regression models. In all models, we will also adjust for potential confounders such as age. When there are missing values, we will explore whether data is missing completely at random (MCAR) or whether data is missing at random (MAR). If MCAR and MAR are satisfied, we will do complete case analysis and explore multiple imputation using Stata software.

Equity effects will be examined by comparing distributions of primary and secondary outcomes between wealth tertiles and educational tertiles within each trial arm. Inequality will also be analysed using the concentration index.

Further details will be provided in a separate statistical analysis plan.

### Qualitative data analysis

The content of the qualitative data from IDIs and FGDs will be explored on the same day as interviews and FGDs are done, and the researchers will adjust the interview guides to enhance their relevance. The transcribed and translated texts will be entered into QSR NVIVO 10. The analysis of the interviews, FGDs, open-ended questions from log forms and observations will follow a classical approach employing Malterud’s ‘Systematic text condensation’ [57], a descriptive and explorative method for thematic cross-case analysis drawing upon Giorgi’s psychological phenomenological analysis. Systematic text condensation consists of the following four steps: (1) total impression; (2) identifying and sorting meaning units; (3) condensation; (4) synthesizing.

### Data monitoring

A Data Monitoring Committee (DMC) with three members has been established. The committee will be independent of the project management team. The committee will advise on study modification or termination based on its reviews of data. A charter for the DMC has been developed and can be obtained from the authors. The DMC will review the follow-up rates from the 6-monthly surveys.

### Benefits and harms

Participants in the intervention arms will benefit from what the intervention packages offer. If the intervention packages are found to have a positive impact and the government consequently introduces a similar programme, adolescents and their families in many communities may benefit.

Unanticipated problems or adverse events such as deaths and hospitalizations and whether these are likely to be related to the trial, will be recorded and reported to the ethics committees (UNZABREC and REK-West) and the Data Monitoring Committee.

### Auditing

All community and parent meetings will be captured photographically if those present consent, and a report form will be filled in that automatically captures GPS coordinates to verify the place and time of the meetings. Youth club and community meetings will be regularly monitored by the research team to observe the quality of the delivery of the intervention.

An external expert assigned by CISMAC will conduct annual monitoring visits during study implementation. If challenges are detected, an action plan to address these will be prepared.

### Adaptions

The intervention will be adaptive, i.e. elements in the intervention package delivered to a cluster will be modified if obstacles to the implementation of the intervention are encountered, the participation is suboptimal or if important changes occur. When the Project Management Team believes it is important to make substantial adaptions of the trial design, CISMAC will be asked to critically consider whether the proposed adaptions are necessary and sufficient. Important protocol modifications will be reported to the DMC, UNZABREC and REK-West, and registered in ISRCTN and clinicaltrials.gov.

### Access to data

The data will be owned by the University of Bergen and the University of Zambia. Project management team members will have access to the data. Other requests for access will be considered after 3 years of trial completion.

### Dissemination plan

This protocol was written following the Standard Protocol Items: Recommendations for Interventional trials (SPIRIT) checklist (see Additional file 10). Dissemination of the research findings will be done through scientific articles in peer-reviewed journals, reports and presentations at national and international academic- and policy-related conferences. We will report findings according to the Consolidated Standards of Reporting Trials (CONSORT) guidelines. Protocol modifications will be reported when disseminating findings. Authorship of scientific articles emerging from the study will be decided upon following guidelines from the International Committee of Medical Journal Editors.

We have established an advisory group with representatives from the MoGE, Ministry of Health (MoH), Ministry of Gender, and the Ministry of Traditional Affairs. We will share and discuss research findings with the group and other key stakeholders in Zambia and the study communities.

## Discussion

### Relevance and potential for impact

This trial will examine the effectiveness of two intervention components that have potential for scale-up and are in line with political priorities in Zambia. A welfare programme in the form of cash transfers is a particularly relevant approach to reduce the effects of poverty as the government is already implementing similar programmes. With payment of schools fees at secondary level, we will mimic what may happen if secondary education becomes free, like the previous government declared that they aimed to achieve [58]. The community component will examine the effects of a SRH curriculum that is in line with the new CSRHE framework from the MoGE and which will be scaled up in all Zambian schools. The programme we will test differs from the government’s CSRHE programme in that it will include youths that are out-of-school, and parents and the wider community will also be engaged in a dialogue around the project’s thematic focus to enhance community support to postpone childbearing and marriage and extend the period of staying in school for girls. A robust evaluation of these interventions is in line with the World Health Organization’s recommendations for research on programmes to prevent early childbearing and marriage [10].

It seems plausible that the bigger the economic support is and the more intense the community intervention is, the stronger the impact may be. However, we have attempted to strike a balance between the scale and intensity of the intervention on the one hand and the wish to establish a programme that is feasible to implement and scale up. The cash transfers that will be offered amount to approximately the same per year as poor families receive through the government’s ‘Social Cash Transfer’ scheme and are likely to be within the range that the government may consider if it were to implement a cash transfer programme for adolescent girls. This amount is estimated to be sufficient to pay for a school uniform and other school materials, and the combination of the cash transfers and payment of school fees will thus make schooling essentially free of cost for the family of an adolescent girl. Regarding the intensity of the community components, we have opted for biweekly meetings to ensure fairly frequent meetings while avoiding adding a huge additional work burden on teachers and CHAs/CHWs.

The sample size calculation has been based on an assumption that the impact of the interventions will be of more or less the same magnitude 2 years after the end of the intervention as at the end of the intervention period. Evaluations of cash transfer programmes have previously indicated that the effects of the cash transfers may continue for a few years after the intervention has come to an end, and in some cases, long-term impact has been found [22]. However, long-term effects may depend on changes in norms taking place [27]. For the community component we expect that it will take some time for community norms to change, but we assume that if normative changes do take place, these will persist for some time after the end of the intervention programme. In fact, it is also possible that the effects strengthen over time, if the interventions initiate a positive process of social and economic change in the communities. However, it is difficult to predict whether this will actually be the case. We have thus included both a primary outcome that will measure the effects on childbirth from pregnancies conceived up to the end of the intervention period, and an outcome that will measure longer-term effects.

Adolescent pregnancy rates are particularly high among girls who have never attended school, and the earlier a girl drops out of school, the higher is the likelihood that she gets pregnant at an early age [49]. We will be recruiting girls enrolled in grade 7 and this obviously implies that we will miss vulnerable girls who do not reach that level of schooling. We have chosen to study the effects of intervening at the transition point between primary and secondary school due to cost and time considerations, but are mindful that we may underestimate the potential effects the interventions could have had if they were implemented at an earlier stage reaching a higher proportion of the most vulnerable participants.

### Bias and confounding

We recognize that adolescence is a period in which many migrate to go to school in other areas, to seek work or to get married [59]. Migration can increase the risk of loss to follow-up of participants, and attrition can reduce the power to detect differences between the study arms, and it can lead to selection bias if those who are lost are different from those who remain in the study. We will attempt to minimize selection bias by providing incentives for follow-up contact interviews and by introducing additional incentives if we experience substantial differential post-randomization dropout, and by including girls who only consent to providing information during follow-up rounds but refuse to receive the intervention. Telephone-based follow-up is likely to be a more feasible method to keep in touch with participants than if we were to track them physically, and contact details will be updated as part of these regular follow-up contacts. In order to incentivize participants to respond to follow-up contacts, replying to these calls will be a prerequisite for receiving the economic support in the intervention arms, and girls in the control arm will be offered a small compensation (of approximately ZMW 20) as a token of appreciation of their time. Girls in the intervention arms will be offered the same compensation after the intervention period is over. We will also invest the necessary resources to physically track the girls if we cannot reach them by phone.

Reporting bias is also a potential risk since all the outcome measurements will be based on self-reports. We hope to minimize recall bias by contacting the participants every 6 months and asking them questions about school enrolment, pregnancy, childbirth and marriage. In addition, some of the self-reported measures can be validated against information from other sources such as school attendance registers and examination records. It is possible that the power to detect differences could be higher for pregnancy-focused measurements than birth measurements since these can capture pregnancies that end in spontaneous or induced abortion. However, many women will avoid reporting pregnancies that do not end in a live birth, and it seems logical that pregnancies are more likely to be under-reported than deliveries as the latter are more difficult to hide. Giving birth may also enhance a girl’s social status. As the primary outcomes should be as valid measures of childbearing as possible, we have selected “given birth” although we recognize that there may be some under-reporting of births that ended with the death of the baby (e.g. stillbirths) as such deaths are associated with stigma [60]. We also risk that some girls may hesitate to report outcomes such as live births, marriage, sexual activity and school dropout due to fear of sanctions since they are aware that the study explicitly aims to prevent these outcomes. Under-reporting is particularly problematic if it is differential between the study arms. We will attempt to reduce social desirability bias by asking sensitive questions towards the end of the interview when some degree of rapport has ideally been achieved, by emphasising that we would like them to give us information on all births, including stillbirths, that there will be full confidentiality, and that there will be no negative implications from the project if the girl drops out of school, gets married or pregnant.

Contamination is a potential risk in relation to the community component. Since some of the schools are only 8 km apart, some communities may have children in both the intervention and the control schools. It may moreover be difficult to prevent guardians and community members from the two other arms from attending community meetings in the combined intervention arm. We will aim to minimize contamination by restricting the youth club to pupils who are enrolled in grade 7 in the combined intervention schools at the start of 2016 and by employing intention-to-treat analysis.

## Conclusions

As far as we are aware, this will be the first cluster RCT to measure the effect of a package combining economic support and a community component to prevent adolescent childbearing in a LMIC. The intervention components have been carefully selected to ensure they will be feasible and sustainable to implement. Increased schooling among adolescent girls is likely to empower them economically [61] and cognitively, and combined with postponed childbearing this can enable them to better protect the health of their children [17] and themselves and moreover increases the probability that their future children will complete secondary school [5]. The findings from this study will be highly relevant for programmes aiming to improve adolescent reproductive health in Zambia and in similar contexts.

### Trial status

The trial was still recruiting participants at the time of submission of the manuscript.

## Additional files

Additional file 1: Information sheet and consent form for parents about the Research Initiative to Support the Empowerment of Girls (RISE). (PDF 425 kb)
Additional file 2: Information sheet and assent form for girls aged < 18 years about the Research Initiative to Support the Empowerment of Girls (RISE). (PDF 426 kb)
Additional file 3: Information sheet and consent form for girls aged ≥ 18 years about the Research Initiative to Support the Empowerment of Girls (RISE). (PDF 427 kb)
Additional file 4: Case record form. (DOCX 25 kb)
Additional file 5: Baseline survey questionnaire 2016. (DOCX 56 kb)
Additional file 6: Parent/guardian questionnaire at time of giving consent. (DOCX 21 kb)
Additional file 7: Field-based follow-up contact questionnaire for control arm. (DOCX 39 kb)
Additional file 8: Field-based follow-up contact questionnaire for economic intervention arm. (DOCX 39 kb)
Additional file 9: Field-based follow-up contact questionnaire for combined intervention arm. (DOCX 40 kb)
Additional file 10: SPIRIT checklists with recommended items to address in a clinical trial protocol and related documents. (DOC 121 kb)

## Abbreviations

CHA: Community health assistant
CHW: Community health worker
CRCT: Cluster randomized controlled trial
CSRHE: Comprehensive Sexual and Reproductive Health Education
DEBS: District Education Board Secretary
DMC: Data Monitoring Committee
FGD: Focus group discussion
FinQ: Final questionnaire
FupQ: Follow-up contact questionnaire
HIV: Human immunodeficiency virus
HSV-2: Herpes simplex virus type 2
IDI: In-depth interview
LMIC: Low- and middle-income country
MoGE: Ministry of General Education
MoH: Ministry of Health
PTA: Parent-Teacher Association
RCT: Randomized controlled trial
SRH: Sexual and reproductive health
STI: Sexually transmitted infection
ZCPH: Zambia Census of Population and Housing

## Footnotes

1 First conducted November 2013, updated in November 2014, using the search query (((pregnancy in adolescence [MeSH]) OR (Adolescent or teen or youth or youths or girls) AND (Pregnancy [MeSH] or pregnant* or birth* or childbirth OR marriage or Marriage [MeSH])) AND ((intervention or program or programme or trial or experiment or experimental) AND (random*) OR (Controlled Clinical Trial [MeSH] or Clinical Trial [MeSH] or Clinical Trial, Phase III [MeSH] or Randomized Controlled Trial [MeSH]))).

2 Medline, Embase, Web of Science, PsycINFO, ERIC, EconLit, Campbell collaboration, Cochrane, CINAHL, POPLINE, LILACS, African Index Medicus, Index Medicus for the Eastern Mediterranean Region, Global Development Finance, Economic Outlook Database, and World Bank e-library.
