# Supplementary material for: Effectiveness of a girls’ empowerment programme on early childbearing, marriage and school dropout among adolescent girls in rural Zambia: study protocol for a cluster randomized trial
Source: Trials. 2016 Dec 9;17:588. doi: 10.1186/s13063-016-1682-9 (PMC5148869; doi:10.1186/s13063-016-1682-9)
Supplement: Additional file 1: — Information sheet and consent form for parents about the Research Initiative to Support the Empowerment of Girls (RISE). (PDF 425 kb) [file 13063_2016_1682_MOESM1_ESM.pdf]

Version Date: 4th June 2016

Information sheet for parents about the Research Initiative to Support the Empowerment of Girls (RISE)

Introduction:

Your daughter is invited to participate in the RISE programme, which is implemented by the University of Zambia. The programmes aims to find the best and most economic way to enhance opportunities for communities to support adolescent girls to continue going to school and increase girls' possibilities to postpone pregnancy and marriage. The programme will test three different ways of enhancing the opportunities of girls and their communities:

- Support package I (30 schools): Material support
- Support package II (60 schools): Material support and economic support
- Support package III (60 schools): Material support , economic support and community dialogues

RISE will be implemented in nine districts: Monze, Mazabuka, Chikankata, Choma, Pemba, Kapiri Mposhi, Chibombo, Kabwe and Mkushi. Girls enrolled in grade seven in 2016 in selected schools will be invited to participate, along with their parents/guardians and their communities. We aim to recruit about 5400 girls and their parents or guardians to be part of the study.

You are being asked to allow your daughter to take part in this research programme because she is a female pupil enrolled in grade 7. Both you and your daughter need to agree in order for your daughter to join. She will need to sign an assent form and you will be asked to sign a consent form. You will be given a copy of this information sheet to keep.

What is the purpose of this research programme?

The aim of the RISE programme is to inform the Government of Zambia how to design future programmes helping adolescent girls to live healthier and more prosperous lives. Three different support packages will be studied containing different combinations of material support, economic support and community dialogues (see later).

A fair procedure to determine which communities get which support package

All communities/schools have equal chances of receiving each of the support packages. Which community gets which package will be determined by a fair lottery where community representatives will be invited to participate. This takes place after communities, girls and guardians have decided to participate in the programme. We will only include schools where

85% or more of the girls in grade 7 and their guardians/parents have agreed to participate in the programme.

What will girls be asked to do in this programme?

Participating girls are expected to allow research assistants to conduct an initial interview with them about their schooling, aspirations for the future and thoughts about pregnancy and marriage. They will also be asked to make themselves available for phone interviews twice a year with representatives from the University of Zambia about schooling, their marital status and childbearing.

In order for us to contact your daughter during the programme period, we will ask her to give us phone numbers she can be contacted via and the name and phone numbers of up to five relatives, neighbours or close friends who can help us find her if the phone she prefers to be contacted via does not work.

There are no other conditions for participation. Girls who do not plan to go to secondary school are also welcome to be part of the programme; and girls who quit school, get married or have children during the programme period can still continue to participate.

What are you as a guardian asked to do in this programme?

You are asked to give permission for your daughter to participate. We also request permission to conduct a very short interview with you immediately after you have signed the consent form to get your contact information, to learn how you are related to this girl, and to record what educational background you and her other guardians have. In addition, we may call you every six months to ask questions about what school, health care and marriage expenses or revenues you have incurred/received related to your daughter.

Programme duration

RISE will start in September 2016 and continue until November 2018. The University of Zambia will contact the participants at 6 months intervals for another two years to learn how they have benefited in terms of their education, health and economic status.

What will happen if my daughter's school ends up in the group receiving…

…Support package I?

She and other enrolled girls will be provided with free writing materials (notebooks, pens and pencils). In addition, every time she is interviewed she will receive a compensation (K30 for the first and last interview in the programme, and K20 for the short 5 minute interviews over the phone). When she is interviewed over the phone she will also be given a lottery ticket to an annual bicycle lottery. The winner will be drawn during an annual ceremony at her school.

…Support package 2?

You will receive a grant (approx. 35 USD) at the beginning of 2017 and 2018 to cover expenses related to your daughter's education (uniforms, shoes, etc.) or to help you support your girl in other ways. She will from September 2016 to November 2018 receive a small monthly allowance of approx. 3-4 USD (through you) that she can spend on small things that

girls need as adolescents, and she will be given free writing materials. School fees will also be paid (up to K 1500 per year) for girls who enrol in grade 8 and 9 in 2017 and 2018.

The only condition she needs to fulfill for her and you receive this economic support is for her to participate in short 5 minute interviews over the phone twice per year. Every time she is interviewed over the phone she will also be given a lottery ticket to an annual bicycle lottery. The winner will be drawn during an annual ceremony at her school. A compensation of K30 will be given for the first and the last interview in the programme.

…Support package 3?

You will receive a grant (approx. 35 USD) at the beginning of 2017 and 2018 to cover expenses related to your daughter's education (uniforms, shoes, etc.) or to help you support your girl in other ways. She will from September 2016 to November 2018 receive a small monthly allowance of approx. 3-4 USD (through you) that she can spend on small things that girls need as adolescents, and she will be given free writing materials. Schools fees will also be paid (up to K 1500 per year) for girls who enrol in grade 8 and 9 in 2017 and 2018.
Your daughter will also be invited to attend a youth club which will cover topics such as the value of education, decision making, communication, early pregnancy and sexual and reproductive health (SRH) training sessions. The meetings will take place every fortnight. Annual meetings will be held with guardians/parents to inform you about the content of the meetings. There will also be community meetings where community members are invited to come to see films and discuss the value of education for adolescent girls, early marriage and early pregnancy.

The only condition she needs to fulfill for her and you receive the economic support is for her to participate in short 5 minute interviews over the phone twice per year. Every time she is interviewed over the phone she will also be given a lottery ticket to an annual bicycle lottery. The winner will be drawn during an annual ceremony at her school. A compensation of K30 will be given for the first and the last interview in the programme.

What will happen if your daughter does not enrol in secondary school or drops out?

If she is in the group receiving support package 2 or 3, she will still receive the monthly transfer and you will receive the support grant until her 18th birthday.

What will happen if your daughter moves or shifts to another school?

Girls who move or shift to another school will also be encouraged to continue to participate. However, they cannot shift group, which means they will continue to be part of the group the school they are enrolled in now will be part of.

What about Confidentiality?

All her personal information will be kept confidential. Your daughter's name will only be saved together with her contact information, and this information will be available to members of the research team only. All other information will be saved using a code number, not names. Only the data manager in Lusaka and the two principal investigators will have access to the information which allows linking of a form ID number to a specific individual. Research records will be stored in a locked room or on secure data servers. Her name will never be used in any publication or presentation about the programme.

At the end of the programme we will collect attendance and examination information about all participating girls from the schools they have been attending, but this information will not be shared with anyone else.

What are the risks and discomforts?

There are no substantial risks or discomforts related to the study. When being interviewed your daughter may refuse to answer any question she finds embarrassing and can stop the interview at any time.

Will the research benefit her or the community?

If your daughter and other girls in this area participate in this study, this will benefit Zambian society and the government since we will gain new knowledge which will help shape future programmes to empower adolescent girls.

No commitments

Participation in the study is voluntary. You and your daughter can withdraw your consent/assent at any time without stating any particular reason. Neither you nor your daughter will be disadvantaged in any way should you decide not to take part or to quit the study at any point. You will not lose your rights to any other care or services that you might have in the community. There will also be no negative consequences to you or your daughter if she drops out of school, gets married or pregnant.

In order to prove that you have been fully informed about the study, we need your signature on the attached consent form for your daughter to participate. You are not making any commitments by signing. If you wish that she participates, please sign the declaration of consent on the final page.

You have the right to ask, and have answered, any questions you may have about this study at any time. If you have questions or concerns, you can contact the following researchers:.

Principal Investigator: Dr Patrick Musonda
Department of Public Health, University of Zambia
Study Contact telephone number: 0963-256-318
Study Contact email: patrick.musonda31@gmail.com
Co-Principal Investigator (Norway): Dr Ingvild Sandoy

All research on human volunteers is reviewed by a committee that works to protect your rights and welfare. If you have questions or concerns about your daughter's rights as a research participant you may contact the University of Zambia Research Ethics Committee. Please phone 01-250-753 to be directed to one of the chairpersons of the committee.

The University of Zambia will run the study in collaboration with three research institutions in Norway: the University of Bergen, the Chr. Michelsen's Institute and the Norwegian School of Economics. The study is funded by the Research Council of Norway.

Version Date: 4th June 2016

PARENTAL/GUARDIAN CONSENT TO ALLOW DAUGHTER TO TAKE PART in the Research Initiative to Support the Empowerment of Girls (RISE)

Name of school: ______________________________

Printed Name of Research Participant: ______________________________________

Participant's Agreement:

I have read or had read to me the information provided above. I have asked all the questions I have at this time. I voluntarily agree to allow my daughter to participate in this research study.

____ ____________________

Signature of Parent/Legal Guardian Date

____

Printed Name of Parent/Legal Guardian

NB! THE PART BELOW ONLY APPLIES IF THE PARENT/GUARDIAN CANNOT WRITE

Ask the parent/guardian to mark a "left thumb impression" in the box
.

____ ____________________

Signature of research assistant Date

____

Printed name of research assistant

____ ____________________

Signature / thumb print of witness (if legal guardian cannot write) Date

______________________________________ ________________________________

Printed Name of Witness Telephone number or address of witness
