# Supplementary material for: Effectiveness of a girls’ empowerment programme on early childbearing, marriage and school dropout among adolescent girls in rural Zambia: study protocol for a cluster randomized trial
Source: Trials. 2016 Dec 9;17:588. doi: 10.1186/s13063-016-1682-9 (PMC5148869; doi:10.1186/s13063-016-1682-9)
Supplement: Additional file 2: — Information sheet and assent form for girls aged < 18 years about the Research Initiative to Support the Empowerment of Girls (RISE). (PDF 426 kb) [file 13063_2016_1682_MOESM2_ESM.pdf]

Version Date: 4th June 2016

Information sheet for girls aged <18 years about the Research Initiative to Support the Empowerment of Girls (RISE)

Introduction

You are invited to participate in the RISE programme, which is implemented by the University of Zambia. The programmes aims to find the best and most economic way to enhance opportunities for communities to support adolescent girls to continue going to school and increase girls' possibilities to postpone pregnancy and marriage. The programme will test three different ways of enhancing the opportunities of girls and their communities:

- Support package I (30 schools): Material support
- Support package II (60 schools): Material support and economic support
- Support package III (60 schools): Material support , economic support and community dialogues

RISE will be implemented in nine districts: Monze, Mazabuka, Chikankata, Choma, Pemba, Kapiri Mposhi, Chibombo, Kabwe and Mkushi. Girls enrolled in grade seven in 2016 in selected schools are being invited to participate, along with their parents/guardians and their communities. We aim to recruit about 5400 girls and their parents or guardians to be part of the study.

You are being asked to to take part in this research programme because you are a female pupil enrolled in grade 7. Both you and your guardians/parents need to agree in order for you to join. You will need to sign an assent form and your guardians/parents will be asked to sign a consent form. You will be given a copy of this information sheet to keep.

What is the purpose of this research programme?

The aim of the RISE programme is to inform the Government of Zambia how to design future programmes helping adolescent girls to live healthier and more prosperous lives. Three different support packages will be studied containing different combinations of material support, economic support and community dialogues (see later).

A fair procedure to determine which communities get which support package

All communities/schools have equal chances of receiving each of the support packages. Which community gets which package will be determined by a fair lottery where community representatives will be invited to participate. This takes place after communities, girls and guardians have decided to participate in the programme. We will only include schools where

85% or more of the girls in grade 7 and their guardians/parents have agreed to participate in the programme.

What will you be asked to do in this programme?

Participating girls are expected to allow research assistants to conduct an initial interview with them about their schooling, aspirations for the future and thoughts about pregnancy and marriage. You will also be asked to respond to some questions over the phone twice a year when representatives from the University of Zambia will call you to ask about your schooling, marital status and childbearing.

In order for us to contact you during the programme period, we will ask you to give us phone numbers you can be contacted via and the name and phone numbers of up to five relatives, neighbours or close friends who can help us find you if the phone you prefer to be contacted via does not work.

There are no other conditions for participation. Girls who do not plan to go to secondary school are also welcome to be part of the programme; and girls who quit school, get married or have children during the programme period can still continue to participate.

Programme duration

RISE will start in September 2016 and continue until November 2018. The University of Zambia will contact the participants at 6 months intervals for another two years to learn how you have benefited in terms of your education, health and economic status.

What will happen if my school ends up in the group receiving…

…Support package I?

You and other enrolled girls will be provided with free writing materials (notebooks, pens and pencils). In addition, every time you are interviewed you will receive a compensation (K30 for the first and last interview in the programme, and K20 for the short 5 minute interviews over the phone). When you are interviewed over the phone you will also be given a lottery ticket to an annual bicycle lottery. The winner will be drawn during an annual ceremony at your school.

…Support package 2?

Your guardians/parents will receive a grant (approx. 35 USD) at the beginning of 2017 and 2018 to cover expenses related to your education (uniforms, shoes, etc.) or to help them support you in other ways. You will from September 2016 to November 2018 receive a small monthly allowance of approx. 3-4 USD (through your guardians/parents) that you can spend on small things that you need as an adolescent, and you will be given free writing materials. School fees will also be paid (up to K 1500 per year) for you if you enrol in grade 8 and 9 in 2017 and 2018.

The only condition you need to fulfill for you and your guardians/parents to receive this economic support is to participate in short 5 minute interviews over the phone twice per year. Every time you are interviewed over the phone you will also be given a lottery ticket to an

annual bicycle lottery. The winner will be drawn during an annual ceremony at your school. A compensation of K30 will be given for the first and the last interview in the programme.

…Support package 3?

Your guardians/parents will receive a grant (approx. 35 USD) at the beginning of 2017 and 2018 to cover expenses related to your education (uniforms, shoes, etc.) or to help them support you in other ways. You will from September 2016 to November 2018 receive a small monthly allowance of approx. 3-4 USD (through your guardians/parents) that you can spend on small things that you need as an adolescent, and you will be given free writing materials. Schools fees will also be paid (up to K 1500 per year) for you if you enrol in grade 8 and 9 in 2017 and 2018.

You will also be invited to attend a youth club which will cover topics such as the value of education, decision making, communication, early pregnancy and sexual and reproductive health (SRH) training sessions. The meetings will take place every fortnight. Annual meetings will be held with guardians/parents to inform them about the content of the meetings. There will also be community meetings where community members are invited to come to see films and discuss the value of education for adolescent girls, early marriage and early pregnancy.

The only condition you need to fulfill for you and your guardians/parents to receive the economic support is to participate in short 5 minute interviews over the phone twice per year. Every time you are interviewed over the phone you will also be given a lottery ticket to an annual bicycle lottery. The winner will be drawn during an annual ceremony at your school. A compensation of K30 will be given for the first and the last interview in the programme.

<u>What will happen if you do not enrol in secondary school or drop out?</u>

If you are in the group receiving support package 2 or 3, you will still receive the monthly transfer and your guardians/parents will receive the support grant until your 18th birthday.

<u>What will happen if you move or shift to another school?</u>

Girls who move or shift to another school will also be encouraged to continue to participate. However, you cannot shift group, which means you will continue to be part of the group the school you are enrolled in now will be part of.

<u>What about Confidentiality?</u>

All your personal information will be kept confidential. Your name will only be saved together with your contact information, and this information will be available to members of the research team only. All other information will be saved using a code number, not names. Only the data manager in Lusaka and the two principal investigators will have access to the information which allows linking of a form ID number to a specific individual. Research records will be stored in a locked room or on secure data servers. Your name will never be used in any publication or presentation about the programme.

At the end of the programme we will collect attendance and examination information about all participating girls from the schools they have been attending, but this information will not be shared with anyone else.

<u>What are the risks and discomforts?</u>
There are no substantial risks or discomforts related to the study. When being interviewed you may refuse to answer any question you find embarrassing and you can stop the interview at any time.

<u>Will the research benefit you or the community?</u>
If you and other girls in this area participate in this study, this will benefit Zambian society and the government since we will gain new knowledge which will help shape future programmes to empower adolescent girls.

<u>No commitments</u>
Participation in the study is voluntary. You and your guardians/parents can withdraw your assent/consent at any time without stating any particular reason. Neither you nor your guardians/parents will be disadvantaged in any way should you decide not to take part or to quit the study at any point. You will not lose your rights to any other care or services that you might have in the community. There will also be no negative consequences to you or your guardians/parents if you drop out of school, get married or pregnant.

In order to prove that you have been fully informed about the study, we need your signature on the attached assent form for you to participate. You are not making any commitments by signing. If you wish to participate, please sign the declaration of assent on the final page.

You have the right to ask, and have answered, any questions you may have about this study at any time. If you have questions or concerns, you can contact the following researchers:.

Principal Investigator: Dr Patrick Musonda
Department of Public Health, University of Zambia
Study Contact telephone number: 0963-256-318
Study Contact email: patrick.musonda31@gmail.com
Co-Principal Investigator (Norway): Dr Ingvild Sandoy

All research on human volunteers is reviewed by a committee that works to protect your rights and welfare. If you have questions or concerns about your rights as a research participant you may contact the University of Zambia Research Ethics Committee. Please phone 01-250-753 to be directed to one of the chairpersons of the committee.

The University of Zambia will run the study in collaboration with three research institutions in Norway: the University of Bergen, the Chr. Michelsen's Institute and the Norwegian School of Economics. The study is funded by the Research Council of Norway.

Version Date: 4th June 2016

ASSENT OF UNDERAGE GIRL TO TAKE PART in the Research Initiative to Support the Empowerment of Girls (RISE)

Name of school: ____________________________

Printed Name of Research Participant: ____________________________________

Participant's Agreement:

I have read or had read to me the information provided above. I have asked all the questions I have at this time. I voluntarily agree to participate in this research study.

____ __________________

Signature of Research Participant Date

NB! THE PART BELOW ONLY APPLIES IF THE PARTICIPANT CANNOT WRITE

Ask the participant to mark a "left thumb impression" in the box

.

____ __________________

Signature of research assistant Date

____

Printed name of research assistant

____ __________________

Signature / thumb print of Witness (if participant cannot write) Date

_____________________________________ ______________________________

Printed Name of Witness Telephone number or address of witness
