# Supplementary material for: Effectiveness of a girls’ empowerment programme on early childbearing, marriage and school dropout among adolescent girls in rural Zambia: study protocol for a cluster randomized trial
Source: Trials. 2016 Dec 9;17:588. doi: 10.1186/s13063-016-1682-9 (PMC5148869; doi:10.1186/s13063-016-1682-9)
Supplement: Additional file 4: — Case record form. (DOCX 25 kb) [file 13063_2016_1682_MOESM4_ESM.docx]

**Case record form**

*Select name from list*

1. **Eligibility screening**

| Female? | (Y/N) |
| --- | --- |
| Enrolled in grade 7? | (Y/N) |
| Attending school selected for RISE-project? | (Y/N) |

1. **Consent issues** (record dates when forms were signed)

| Date of parental consent |  |
| --- | --- |
| Date of girl’s assent |  |

1. **Date and time** of filling out this form**_ _/_ _/_ _**
2. **Contact information**

| Age |  | |
| --- | --- | --- |
| Do you know your exact date of birth? | Yes/no | |
| If yes, date of birth | __/__/___ | |
| If no, enter month and year |  | |
| What is the name of the village or locality where you live? |  | |
| Mention one landmark (such as church, mosque, market, health post, clinic, hill, etc.) close to your home. |  | |
| Mention a well-known person living close to your home. | First name  Last name | |
| *A household is a group of people who live together and have meals together and they have one person they identify as head.*  What are the official name of the head of your household? | First name Last name | |
| What is the relationship of the head of the household to you? | MotherFatherStepmotherStepfatherSisterBrotherUncleAunt | GrandmotherGrandfatherHusbandCousinOther, specify |
| Do you have a phone? | Yes/no | |
| What is the number of your mobile phone? | _ _ _ _ _ _ _ _ | |

1. Other mobile phone numbers

In this study we need to make phone calls to follow-up where you are staying and what you are doing (e.g. attending school, working), and whether you are married and have children. We will call you twice per year to ask you a few questions.

| Mention 5 alternative phone numbers where you can be reached when we need to contact you via phone. Specify whose numbers these are (e.g. uncle, aunt, brother, sister, friend, neighbour) and the village or locality of this person |
| --- |
| \|  \| Number \| What is the relationship of this person to you? Mother, father, stepmother, stepfather, sister, brother, uncle, aunt, grandmother, grandfather, husband, cousin, neighbour, other \| What is the first name of this person? What is the last name of this person? \| Where does this person live (village or locality) \| \| --- \| --- \| --- \| --- \| --- \| \| 1 \|  \|  \|  \|  \| \| 2 \|  \|  \|  \|  \| \| 3 \|  \|  \|  \|  \| \| 4 \|  \|  \|  \|  \| \| 5 \|  \|  \|  \|  \| |

1. When we call you, we need to be able to verify that we are talking to the right person. Thus we will ask you three control questions. Please tell me what the correct responses to these questions are:
2. i. “What is the first name of your oldest cousin?____________________________

ii. “What is the last name of your oldest cousin? __________________________

If does not have a cousin, enter participant’s own name.

B) What is your middle name? _____________________

If no middle name, enter “NONE”

C) What was your mother’s last name before she married? _________________

If mother not yet married, note current name. If respondent does not know mother’s maiden name, record the girl’s family name.

*GPS coordinates must be captured before closing the form*

**Thank you very much!**

*Please proceed to baseline interview questionnaire.*
