# Supplementary material for: Effectiveness of a girls’ empowerment programme on early childbearing, marriage and school dropout among adolescent girls in rural Zambia: study protocol for a cluster randomized trial
Source: Trials. 2016 Dec 9;17:588. doi: 10.1186/s13063-016-1682-9 (PMC5148869; doi:10.1186/s13063-016-1682-9)
Supplement: Additional file 5: — Baseline survey questionnaire 2016. (DOCX 56 kb) [file 13063_2016_1682_MOESM5_ESM.docx]

Enquiries: Dr. Patrick Musonda

Mobile Number 0963256318

**CISMAC BASELINE SURVEY QUESTIONNAIRE 2016**

**Instructions for interviewer/supervisors:**

1. This interview will be conducted immediately after the Case record form has been filled in
2. Inform the participant that this is not a test and that there are no right or wrong answers. Ask the participant to be honest in her answers, and not to give answers that she thinks we want. We need to know what young people really think to develop the best programmes to empower girls.
3. Do not read response options to the respondent unless the question tells you to do so.
4. Remember to use your interview skills.

- Study ID number
- Date and time of data collection *(should be entered automatically)*
- *Name of District and school should be shown automatically when ID number is entered*

Any information you give me during this interview will be kept confidential, and your name will not be recorded together with this information. So please be honest when you respond.

**SECTION A: DEMOGRAPHIC DATA**

1. How many persons living in your household* are below 18 years?

**A household is a group of people who live together and have meals together and they have one person they identify as head. Persons who are temporarily away, such as at boarding school, should also be counted.*

1. How many persons living in your household are 18 years or above?
2. Are any of your biological parents living with you?

- Yes, mother
- Yes, father
- No

1. Is your biological mother alive? *(skip if living with biological mother)*

- Yes
- No

1. Is your biological father alive? *(skip if living with biological father)*

- Yes
- No

1. What is the main material of the floor in your house?

- Natural floor (earth, sand, dung)
- Rudimentary floor (wood planks, bamboo)
- Finished floor (parquet or polished wood, vinyl, ceramic tiles, concrete cement, carpet)

1. What is the main material of the roof of your house?

- Natural roofing (no roof, thatch, palm leaf)
- Rudimentary roofing (rustic mat, palm/bamboo, wood planks, cardboard)
- Finished roofing (metal/iron sheets, wood, calamine/cement fibre/asbestos, ceramic tiles, cement roofing shingles, mud tiles)

1. Do you have any of the following in your home?

a) Mobile phone

- *Yes*
- *No*

b) Radio

- *Yes*
- *No*

c) Television

- *Yes*
- *No*

d) Refrigerator

- *Yes*
- *No*

e) Electricity

- *Yes*
- *No*

f) Bicycle

- *Yes*
- *No*

g) Plough

- *Yes*
- *No*

h) Animal-drawn cart

- *Yes*
- *No*

*i) Cattle*

- *Yes*
- *No*

*If no to 8i, skip q9*

1. How many cattle does your household have? ___
2. Does any member of your household own# any agricultural land?

- *Yes*
- *No*

#Includes customary land

1. These days, would you say that this household usually has enough food to eat, sometimes has enough food to eat, seldom has enough food to eat, or never has enough food to eat?
   - Usually/always
   - Sometimes
   - Seldom
   - Never
2. How many meals, excluding snacks, do you normally have in a day? __
3. In the last one week, did you or any member of your household have to go to bed hungry because you didn’t have enough food to eat?

- *Yes*
- *No*

1. Last week, did you use
   - Lotion

- *Yes*
- *No*
  - Soap
- *Yes*
- *No*
  - Make-up
- *Yes*
- *No*

*If no to 14 a-c, skip q 15*

1. If you used any of the items above, who paid for this?
   - Your father
   - Your mother
   - Someone else in the family
   - Your boyfriend
   - Yourself
   - Other, specify __________________
2. Who do you ask if you need money?
   - Your father
   - Your mother
   - Someone else in the family
   - Your boyfriend
   - No one
   - Other, specify __________________
3. If there is a need to provide a clarification, please add it here:

**SECTION B: EDUCATION BACKGROUND/DATA**

1. How old were you when you first enrolled in grade 1? __ years
2. Have you repeated any grade?

- *Yes*
- *No*

1. What kind of transport did you use to come to school today? *If did not attend school today, ask about the last time she attended school.*
   - Walk
   - Cycle
   - Bus
   - Motorcycle
   - Car
   - Other. Specify_____________
2. How long did it take you to get to school from your home today (in minutes)? *If did not attend school today, ask about the last time she attended school.*

…………………………………….

1. Are you a boarder?
   - Yes
   - No *(If No, skip the next two questions )*
2. What type of a boarder are you?
   - Weekly
   - Monthly
   - Termly
3. Is there an adult supervising the boarding facility?

- Yes
- No

1. How far do you expect that you will go in your education?
   - Complete grade 7
   - Complete grade 9
   - Complete grade 12
   - Go to college or university
   - Don’t know
2. If you had enough money to pay school fees and could choose freely, how far would you wish to go in your education?
   - Complete grade 7
   - Complete grade 9
   - Complete grade 12
   - Go to college or university
   - Don’t know

**We would like to know whether you agree or disagree with the following statements about education. Do you agree very much, agree, neither agree nor disagree, disagree, or disagree very much with the statements I will read?**

1. My mother thinks that it is important for me to continue to junior secondary school and complete grade 9.

- *1 Strongly agree/agree very much*
- *2 Agree*
- *3 Neither agree nor disagree*
- *4 Disagree*
- *5 Strongly disagree/disagree very much*
- *Does not have a mother*

1. My father thinks that it is important for me to continue to junior secondary school and complete grade 9..

- *1 Strongly agree/agree very much*
- *2 Agree*
- *3 Neither agree nor disagree*
- *4 Disagree*
- *5 Strongly disagree/disagree very much*
- *Does not have a father*

1. If I complete grade 9, I will significantly increase my future income.

- *1 Strongly agree/agree very much*
- *2 Agree*
- *3 Neither agree nor disagree*
- *4 Disagree*
- *5 Strongly disagree/disagree very much*

1. If I complete grade 9, I will benefit even if it doesn’t increase my future income.

- *1 Strongly agree/agree very much*
- *2 Agree*
- *3 Neither agree nor disagree*
- *4 Disagree*
- *5 Strongly disagree/disagree very much*

**SECTION C: KNOWLEDGE, BELIEFS AND NORMS TO PREGNANCY, MARRIAGE, CONTRACEPTIVES AND STIs**

**Below are some statements on pregnancy, family planning and sexually transmitted infections. Are these statements correct or not correct in your opinion?**

**Knowledge**

1. Young girls who use contraceptive pills or injections are at risk of becoming infertile.

- *Yes/Correct*
- *No/Not correct*
- *I don’t know*

1. A girl can get pregnant if she has unprotected sex with a boy three days after the end of her menstrual period.

- *Yes/Correct*
- *No/Not correct*
- *I don’t know*

1. When a girl uses contraceptive pills or the injection for family planning, this protects her against sexually transmitted infections (STI).

- *Yes/Correct*
- *No/Not correct*
- *I don’t know*

**Beliefs and norms**

**We would like to know whether you agree or disagree with the following statements about education. Do you agree very much, agree, neither agree nor disagree, disagree, or disagree very much with the statements I will read?**

1. In my school, most learners my age have had sexual intercourse.

- *1 Strongly agree/agree very much*
- *2 Agree*
- *3 Neither agree nor disagree*
- *4 Disagree*
- *5 Strongly disagree/disagree very much*

1. In my school, most learners do not use a condom if they have sexual intercourse.

- *1 Strongly agree/agree very much*
- *2 Agree*
- *3 Neither agree nor disagree*
- *4 Disagree*
- *5 Strongly disagree/disagree very much*

1. Girls below 18 years of age who have a child are treated with more respect than girls below 18 years who do not have a child.

- *1 Strongly agree/agree very much*
- *2 Agree*
- *3 Neither agree nor disagree*
- *4 Disagree*
- *5 Strongly disagree/disagree very much*

1. Girls below 18 years of age who have a child are a significant economic burden to their family.

- *1 Strongly agree/agree very much*
- *2 Agree*
- *3 Neither agree nor disagree*
- *4 Disagree*
- *5 Strongly disagree/disagree very much*

1. Overall it is be better for girls to have a child before their 18^th^ birthday than to wait until later.

- *1 Strongly agree/agree very much*
- *2 Agree*
- *3 Neither agree nor disagree*
- *4 Disagree*
- *5 Strongly disagree/disagree very much*

1. My mother would strongly disapprove if I became pregnant now.

- *1 Strongly agree/agree very much*
- *2 Agree*
- *3 Neither agree nor disagree*
- *4 Disagree*
- *5 Strongly disagree/disagree very much*
- *Does not have a mother*

1. My father would strongly disapprove if I became pregnant now.

- *1 Strongly agree/agree very much*
- *2 Agree*
- *3 Neither agree nor disagree*
- *4 Disagree*
- *5 Strongly disagree/disagree very much*
- *Does not have a father*

1. My neighbours would strongly disapprove if I became pregnant now.

- *1 Strongly agree/agree very much*
- *2 Agree*
- *3 Neither agree nor disagree*
- *4 Disagree*
- *5 Strongly disagree/disagree very much*

1. If I become a mother before my 18^th^ birthday, adults will treat me with more respect.

- *1 Strongly agree/agree very much*
- *2 Agree*
- *3 Neither agree nor disagree*
- *4 Disagree*
- *5 Strongly disagree/disagree very much*

1. If I become a mother before my 18^th^ birthday, I will become a significant economic burden to my family.

- *1 Strongly agree/agree very much*
- *2 Agree*
- *3 Neither agree nor disagree*
- *4 Disagree*
- *5 Strongly disagree/disagree very much*

1. Overall it will be better for me if I have a child before I am 18 than to wait until later.

- *1 Strongly agree/agree very much*
- *2 Agree*
- *3 Neither agree nor disagree*
- *4 Disagree*
- *5 Strongly disagree/disagree very much*

1. Girls below 18 years of age who are married are treated with more respect than girls below 18 years who are not married.

- *1 Strongly agree/agree very much*
- *2 Agree*
- *3 Neither agree nor disagree*
- *4 Disagree*
- *5 Strongly disagree/disagree very much*

1. My neighbour approve when girls below 18 years of age get married.

- *1 Strongly agree/agree very much*
- *2 Agree*
- *3 Neither agree nor disagree*
- *4 Disagree*
- *5 Strongly disagree/disagree very much*

**SECTION D: MARITAL STATUS**

**I now have some questions about your marital status**

1. Are you married?

- *Yes*
- *No*

*Skip q48-50 if said yes to q47.. Skip q51 if no to y 47.*

1. My mother would like me to get married within the next 3 years

- *1 Strongly agree/agree very much*
- *2 Agree*
- *3 Neither agree nor disagree*
- *4 Disagree*
- *5 Strongly disagree/disagree very much*
- *Does not have a mother*

1. My father would like me to get married within the next 3 years

- *1 Strongly agree/agree very much*
- *2 Agree*
- *3 Neither agree nor disagree*
- *4 Disagree*
- *5 Strongly disagree/disagree very much*
- *Does not have a father*

1. My neighbours would approve if I get married within the next 3 years

- *1 Strongly agree/agree very much*
- *2 Agree*
- *3 Neither agree nor disagree*
- *4 Disagree*
- *5 Strongly disagree/disagree very much*

1. If yes, how old were you when you first started living with your husband? ……. Years
2. *If no to q56*:At what age do you want to get married?__

*If said yes to q46, skip q 53 and 54.*

1. Have you ever had a boyfriend?

- *Yes*
- *No*

*If no, skip q54*

1. Do you currently have a boyfriend?

- *Yes*
- *No*

**Now we will ask you some questions about your behaviour. Please answer** **honestly. Remember that no one at your school or home will know your answers.**

1. Have you ever given birth?

- Yes
- No

*If no to q55, skip q 56 and 57.*

If yes, on which date and in which year did you give birth? ____

Does not remember exact date

56.b If does remember exact date, enter month and year ____________

1. How many children have you given birth to in total in your life (include both those who are alive and those who have died)?

………………

*If yes to q55, skip q58*

1. Have you ever been pregnant?

- *Yes*
- *No*

1. Are you currently pregnant?

- *Yes*
- *No*
- *I don’t know*

1. If yes, how old were you when you first became pregnant? ……. years
2. Have you ever used a contraceptive method, e.g. condom?

- *Yes*
- *No*

*If no to q61, skip q 62*

1. The last time you used a contraceptive method, where did you get it?
   - Government hospital
   - Government health centre/post
   - Mobile clinic
   - Community-based distributor
   - Private hospital/clinic
   - Pharmacy
   - Mission hospital/clinic
   - Shop.
   - Friends/relatives

**Please tell us your views about the following statements even if you have never tried to obtain a condom or other contraceptive.**

1. If you needed a contraceptive, e.g. a condom, how easy or difficult would it be for you to obtain one: very easy, easy, neither easy nor difficult, difficult, or very difficult?

- *1 Very easy*
- *2 Easy*
- *3 Neither easy nor difficult*
- *4 Difficult*
- *5 Very difficult*

1. I would be able to go to a clinic to fetch condoms

- *1 Strongly agree/agree very much*
- *2 Agree*
- *3 Neither agree nor disagree*
- *4 Disagree*
- *5 Strongly disagree/disagree very much*

1. I would be able to go to a pharmacy or a shop to buy condoms

- *1 Strongly agree/agree very much*
- *2 Agree*
- *3 Neither agree nor disagree*
- *4 Disagree*
- *5 Strongly disagree/disagree very much*

*Capture GPS coordinates*

Thank you very much for answering my questions. Let me mention again that all the information you have given me will be kept confidential, and your name will not be recorded together with this information. Thank you very much for participating in this interview and this study.
