# Supplementary material for: Effectiveness of a girls’ empowerment programme on early childbearing, marriage and school dropout among adolescent girls in rural Zambia: study protocol for a cluster randomized trial
Source: Trials. 2016 Dec 9;17:588. doi: 10.1186/s13063-016-1682-9 (PMC5148869; doi:10.1186/s13063-016-1682-9)
Supplement: Additional file 6: — Parent/guardian questionnaire at time of giving consent. (DOCX 21 kb) [file 13063_2016_1682_MOESM6_ESM.docx]

**Parent/guardian questionnaire at time of giving consent**

1. Name of daughter who is eligible to participate in the RISE project: _________________
2. Name of parent/guardian: __________________
3. What is the name of the village or locality where you live? ______________________
4. What is the number of your mobile phone? _ _ _ _ _ _ _ _

- Does not have a phone

1. What is your relationship to _____?

# Mother

# Father

# Stepmother

# Stepfather

# Sister

# Brother

# Uncle

# Aunt

# Grandmother

# Grandfather

# Husband

# Cousin

# Other, specify

1. Have you ever attended school?

- Yes
- No

Skip question 7 if never attended school.

1. What is the highest level of school you attended?

- Lower primary
- upper primary
- Lower/junior secondary
- Upper/senior secondary
- Higher than secondary

1. Are you the head of the household in which __________ lives?

- Yes
- No

If yes, skip question 9.

1. What is the highest level of school attended by the head of the household where _________lives?

- Lower primary
- upper primary
- Lower/junior secondary
- Upper/senior secondary
- Higher than secondary
- Don’t know

If the respondent is the mother of an eligible participant, skip question 10:

1. What is the highest level of school ____’s biological mother attended?

- Never attended
- Lower primary
- upper primary
- Lower/junior secondary
- Upper/senior secondary
- Higher than secondary
- Don’t know

If the respondent is the father of an eligible participant, skip question 11:

1. What is the highest level of school ____’s biological father attended?

- Never attended
- Lower primary
- upper primary
- Lower/junior secondary
- Upper/senior secondary
- Higher than secondary
- Don’t know

In this study we want to contact the girls who participate every six months to follow-up where they are staying, what they are doing (e.g. attending school, working), and whether they are married and have children. If we cannot reach you or your daughter via your phone, is there anyone else we can contact to get in touch with you and your daughter?

| Mention 5 alternative phone numbers when we need to contact your daughter via phone. Specify whose numbers these are (e.g. uncle, aunt, brother, sister, friend, neighbour) and the village or locality of this person |
| --- |
| \|  \| Number \| What is the relationship of this person to your daughter? \| What is the first and last name of this person? \| Where does this person live (village or locality) \| \| --- \| --- \| --- \| --- \| --- \| \| 1 \|  \|  \|  \|  \| \| 2 \|  \|  \|  \|  \| \| 3 \|  \|  \|  \|  \| \| 4 \|  \|  \|  \|  \| \| 5 \|  \|  \|  \|  \| |

Thank you very much for this information and for giving permission for _________ to participate in this project!
