# Supplementary material for: Effectiveness of a girls’ empowerment programme on early childbearing, marriage and school dropout among adolescent girls in rural Zambia: study protocol for a cluster randomized trial
Source: Trials. 2016 Dec 9;17:588. doi: 10.1186/s13063-016-1682-9 (PMC5148869; doi:10.1186/s13063-016-1682-9)
Supplement: Additional file 9: — Field-based follow-up contact questionnaire for combined intervention arm. (DOCX 40 kb) [file 13063_2016_1682_MOESM9_ESM.docx]

**Field-based follow-up contact questionnaire for combined intervention arm**

Research Assistant Name:­­­­­­­­­­­­­­ ____________________________

Date of Interview: __________________________________

**Before we start I need to ask you some questions to make sure I am talking to the right person**

*The correct response will appear in the form.*

A)

1. What is your date of birth? (day-month-year)
2. Correct response?
   - Yes
   - No

*If the response is correct, the next 3 questions can be dropped*

B)

1. What is the name of your oldest cousin?_____
2. Correct response?
   - Yes
   - No

C)

1. What was your mother’s last name before she married? _________________
2. Correct response?
   - Yes
   - No

**D)**

What is your middle name? _____

1. Correct response?
   - Yes
   - No

*If incorrect responses are given to 2 or more control questions, confirm with the head teacher or the parents what the correct responses should be before you proceed.*

1. Do you live in the same place as you did the last time you were interviewed or have you moved to a different place?

- - Lives in the same place
  - Has moved

*If she lives in the same place, skip q2 -5*

1. What is the name of the village where you live? _____________________
2. What is the name of the district where you live?

_______________________

1. Please mention a special landmark or popular place close to your house that can guide someone who does not know where you live?

__________________________

**Personal Information**

1. Are you currently going to school?
   - Yes
   - No

*If no, skip questions 6 and 7.*

1. Which school are you enrolled in? _______________
2. How many days did you go to school last week?

*If no to q 5 or zero to q7, ask q 8*

1. When did you last attend school? ______________________(Response format: mm/yyy)

**I now have some questions about your experiences with the programme**

1. In the last 6 months, did you receive the monthly cash transfer from the project?
   - Yes, every month
   - Yes, some months
   - No
   - Don’t know

*If no, skip question 10-12*

1. If yes, the last time you received the monthly transfer, how much money did you receive? ___________________
2. Did you give anything of the monthly transfer to your guardians/parents or did you keep all of it to yourself?

- Gave to parents/guardians
- Kept all of it

1. How much did you give to your guardians/parents
2. Have you participated in any of the youth club meetings over the last 6 months?
   - Yes
   - No

*If no to q13, skip q14 and 15.*

1. Is there anything in particular you remember from the meetings?
2. How many youth club meetings have you attended in the last school term?

__________ meetings

We would like to learn from you how you experience being part of the RISE project.

1. Have you experienced any good things as a result of your participation in this RISE project?
   - Yes
   - No *->skip q17*
2. What have you experienced?
3. Have you experienced any problems as a result of your participation in the RISE project?
   - Yes
   - No *-> skip q19*
4. What have you experienced?

**Information about health care utilization**

1. Have you been admitted to the clinic or hospital in the last 6 months?

- yes
- No

*If no, skip q21 and 22*

1. Were you admitted due to any female issues?
   - yes
   - No
2. Were you admitted due to pregnancy or birth related issues?
   - yes
   - No
3. Have you visited the outpatient clinic or pharmacy/drug shop or traditional birth attendant or healer in the last 6 months?

- yes
- No

*If no, skip q24 and 25*

1. Was your visit to these places or people due to any female issues?

- yes
- No

1. Was your visit to these places or people due to pregnancy or birth related issues?

- yes
- No

1. Are you married or living with a boyfriend?

- Yes
- No-> skip q27

1. When did you get married or start living with your boyfriend? Month _____ Year ______
2. Have you ever given birth?
   - Yes
   - No -> skip 29-38
3. When did you last give birth? ___(Date or month and year )

*Skip this section if last gave birth before the last interview conducted.*

**Information about the occurrence of birth complications among girls who have recently given birth (within the past 6 months?)**

1. Was the baby born more than one month before the due date?

- Yes
- No
- Don't know

*If answers yes, ask q31.*

1. Can you estimate how many months pregnant were when you gave birth, based on your last menstrual period?---------------months
2. How much did the baby weigh when he/she was born? ___

- Don’t know

*If she does not know the exact weight ask, q33, otherwise skip it:*

1. Did the baby weigh less than 2500 g when he/she was born?

- Yes
- No
- Don't know

1. Is the baby alive?
   - Yes
   - No
2. Did you receive treatment for high blood pressure while you were pregnant?

- Yes
- No

*If no, skip q36*

1. Were you diagnosed with hypertension before you got pregnant

- Yes
- No

1. Did you have any other complications?
2. Mention any other birth complication for which you received treatment? What happened?

- -------------------------------
- ........................................
- ........................................
- ........................................

1. A) We need to make sure we have updated contact information. Which mobile phone number should we use when we call you in the future? _______________________________

B)Whose number is this?______________________

1. A) Can you give us 2 other numbers that we can reach you on in case we cannot reach you on this number?
   - Yes-> 40B
   - Don’t know other phone numbers -> 40F

B) Provide first number ______________________________

C) Whose number is this?______________________

D) Provide second number_______________________________

E) Whose number is this?______________________

40 F *If the girl is interviewed at home:* After you and I have finished talking, may I ask your guardians for 2 alternative phone numbers we may try if we cannot get through on the numbers we have next time we call?

- Yes
- No

This is the end of the interview, thank you for taking time to answer my questions. We are very grateful. Your participation is extremely important for this project because it gives us an opportunity to understand how girls who receive the different support packages fare.

Do you have any questions to me before we finish the interview?

As an appreciation of your collaboration, we will give you a ticket for a bike lottery which will be held at your school in July. You will receive further information on when the draw will take place.

Thank you very much again! We will contact you again in 4-6 months from now.

*If yes to 40 A, end interview here*

1. I*f the girl is interviewed at home:* Now I would like to ask your guardians for some additional phone numbers

Thanks a lot for allowing us to speak to your daughter. I would like to find out whether you are able to give us 2 other numbers that we can reach your daughter on in case we cannot reach you or her on this number when we call next time in 4-6 months?

A) Provide first number______________________________

B) Whose number is this?______________________

C) Provide second number_______________________________

D) Whose number is this?______________________

Thank you so much! We will call to speak to your daughter in 4-6 months. Thanks a lot for your assistance!

*If adverse events are reported, the research assistant should let the data manager know so that he can consider whether there is a need for the project to take action because of the adverse events reported.*

Capture Location
